# p53: a tumor suppressor hiding in plain sight

**DOI:** 10.1093/jmcb/mjz068

**Published:** 2019-08-16

**Authors:** Suzanne J Baker, Bert Vogelstein

**Affiliations:** 1 St Jude Children’s Research Hospital, Department of Developmental Neurobiology, 262 Danny Thomas Place, Memphis, TN 38105, USA; 2 Ludwig Center & Howard Hughes Medical Institute, Johns Hopkins Kimmel Cancer Center, 1650 Orleans Street St, Baltimore, MD 21205, USA

It is a pleasure to join the other authors in this issue to honor Dr Arnold Levine and the remarkable impact of his research. Our paths crossed with Arnie’s when our genetic analyses of colorectal cancers led us to investigate the p53 gene and identify it as a tumor suppressor gene. p53 was initially identified by Arnie and others as an oncogene and tumor antigen, nearly a decade before its role as a tumor suppressor gene was revealed. We fondly remember Arnie visiting our lab, and we visiting his, to discuss the implications of the genetic alterations our group identified in human cancers and how we could work together to investigate the voluminous new questions these mutations raised.

## The rationale for the existence of tumor suppressor genes

The hunt for tumor suppressor genes was a high-risk endeavor in the 1980s. Somatic cell hybridization studies provided early evidence of the existence of tumor suppressor genes by showing that tumorigenic growth was a recessive trait. Hybrid cell fusions of malignant cells with non-malignant cells could suppress tumorigenic growth, and microcell mediated-transfer of specific human chromosomes could suppress the tumorigenic growth of human cancer cell lines in immunodeficient mice (Harris et al., 1969; Klinger, 1982; Peehl and Stanbridge, 1982; Harris, 1988). Alfred Knudson’s insightful analyses of retinoblastoma incidence connected the concept of tumor suppressor genes with human disease pathogenesis (Knudson, 1971, 1985). What later became known as the ‘two-hit hypothesis’ proposed that patients with familial retinoblastoma inherited the first ‘hit’, an inactivating mutation in one allele of a tumor suppressor gene that predisposed them to the disease. A somatic mutation, or second ‘hit’, in the remaining allele would result in complete loss of function of the gene and contribute to tumorigenesis. In sporadic tumors, both hits were hypothesized to occur within the same somatic cell. Due to the much lower likelihood of two somatic mutations, sporadic retinoblastomas were typically unilateral and arose at a later age.

Cytogenetic abnormalities and submicroscopic deletions of chromosome 13q14 revealed the chromosomal location of the putative tumor suppressor gene in retinoblastoma (Francke and Kung, 1976; Knudson et al., 1976; Benedict et al., 1983; Cavenee et al., 1983, 1985; Godbout et al., 1983). These studies later led to the positional cloning of the RB1 gene, thus validating the two-hit hypothesis (Friend et al., 1986; Fung et al., 1987; Lee et al., 1987).

## Mapping the chromosome 17p tumor suppressor locus

In the late 1980s, our group mapped regions of chromosomal loss in colorectal cancer to identify the locations of tumor suppressor genes. The highest frequency loss involved the short arm of chromosome 17 (17p), which occurred in >75% of colorectal carcinomas (Reichmann et al., 1981; Muleris et al., 1985; Fearon et al., 1987; Vogelstein et al., 1988). Sporadic colorectal cancers posed a significant challenge compared to hereditary cancer predisposition syndromes like retinoblastoma where small constitutional deletions narrowed down the target area for analysis. To more precisely localize the candidate tumor suppressor, we performed Southern blots with a panel of 20 different polymorphic markers on 17p to evaluate loss of heterozygosity (LOH) in 58 paired samples of colorectal carcinoma and matched normal colorectal tissues. This analysis identified a minimal common region of deletion shared among all tumors in which any LOH was observed. This region encompassed approximately half of 17p (Baker et al., 1989). With today’s genomic maps, we can estimate that the common region of deletion spanned >12.5 megabase pairs of DNA and contained ~577 genes, including 480 protein-coding genes. Relative to today, genomic maps in 1988 were extremely sparse and much of the genome could be considered uncharted territory in terms of the density and identity of genes. Identifying the tumor suppressor gene within this area was a daunting prospect, and we did not consider it likely when we started this project in the mid-80’s that the gene could actually be identified within a time-frame consistent with a pre-doctoral thesis. Remember that at the time (1985), oncogenes were already known but tumor suppressor genes were mythical beasts, predicted to exist but not yet sighted.

## *p53*: oncogene or tumor suppressor gene

p53 was identified in 1979 by Arnie Levine’s group, as well as four other groups, as a host protein bound to large T antigen in cells infected with the transforming virus SV40 (Kress et al., 1979; Lane and Crawford, 1979; Linzer and Levine, 1979; Melero et al., 1979; Smith et al., 1979), and by another group as a transformation-related antigen in chemically induced mouse sarcomas (DeLeo et al., 1979). Multiple lines of research initially supported the hypothesis that *p53* was an oncogene, including association of the p53 protein with viral transforming proteins of SV40 or adenovirus in infected cells, and elevated expression of p53 protein in transformed cells and human tumor cell lines. The *p53* cDNA was cloned by several groups, often from tumor cell lines with robust p53 protein expression, and expression of *p53* cDNA could cooperate with other oncogenes to transform primary mouse cells and increase tumorigenic growth of established tumor cells. While multiple lines of evidence supported the widespread view that *p53* was an oncogene, there were some observations that had been interpreted as suggesting a more complex story (Wolf and Rotter, 1985; Eliyahu et al., 1988; Finlay et al., 1988; Hicks and Mowat, 1988; Hinds et al., 1989). One explanation for these complexities was that p53 was actually a tumor suppressor gene rather than an oncogene. This interpretation was largely based on the fact that the normal p53 gene from mice inhibited cell growth. The investigators were appropriately cautious about this interpretation; we now know that many oncogenes, such as BCL2 and IDH1, when overexpressed, can inhibit growth (Vogelstein et al., 2013).

## Testing the two-hit hypothesis

We began our studies of p53 in 1987, assuming that it was an oncogene, for the reasons described above. In particular, we initially did not think that it was the tumor suppressor gene target of the chromosome 17p deletions for which we were searching. But because the p53 gene was in the middle of the ‘lost chromosome region’ on chromosome 17p in colorectal cancers, and because it had been implicated to play a role in neoplasia, we thought we had to eliminate it as a candidate before continuing the search for the actual tumor suppressor gene on chromosome 17p. Based on the two-hit hypothesis, we began our evaluation by looking for evidence of homozygous deletion or rearrangements of the *p53* locus in colorectal carcinomas; none were detected by Southern blot analysis. Northern blot analysis of colorectal carcinomas showed that *p53* was expressed in most colorectal carcinomas, and there was no evidence of abnormally sized transcripts. To test for more subtle alterations in *p53*, we selected a single tumor that had an allelic deletion of chromosome 17p and sequenced the remaining *p53* allele in this tumor (Baker et al., 1989). To our initial disbelief, it contained a *p53* missense mutation in the remaining allele at codon 143 (V143A). This change was not present in the normal colon mucosa of this patient, so it was somatic, in accordance with the two-hit hypothesis. But a change from valine to alanine did not necessarily mean that the encoded protein would be drastically different, and we wished to confirm this observation in a second colorectal cancer in which one chromosome 17p allele was lost. The second tumor did indeed contain a p53 mutation, this time at codon 175 (R175H).

**Figure 1:**
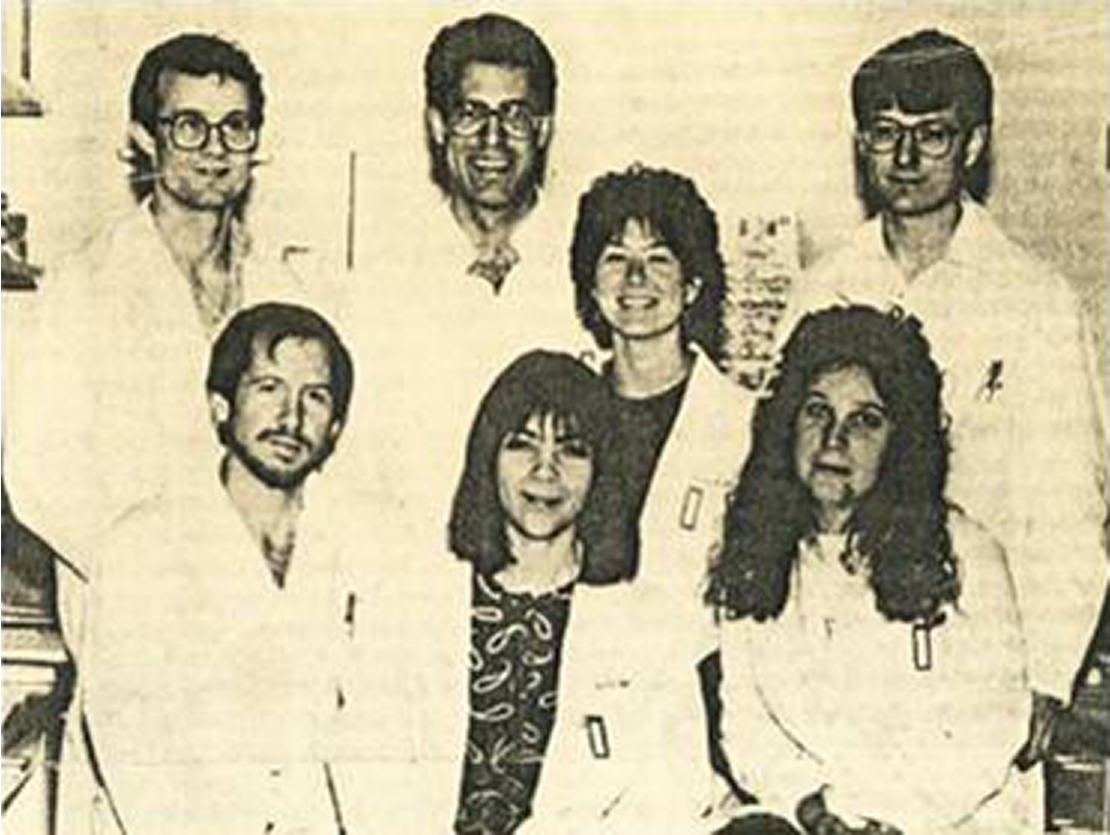
Members of the Vogelstein Lab, circa 1989. Front row: Bert Vogelstein, Janice M. Nigro, Ann C. Preisinger; middle row: Suzanne J. Baker; back row: J. Michael Ruppert, Eric R. Fearon, Kenneth W. Kinzler.

Finally, we showed that these types of genetic alterations were not confined to colorectal cancers. Working with Janice Nigro and others in our lab (Figure 1), we found that 17p allelic losses coupled with missense mutations in the remaining *p53* allele occurred in many tumors of the brain, breast, lung, and mesenchyme, in addition to a larger cohort of colorectal cancers (Nigro et al., 1989; Baker et al., 1990). These studies also showed that there were ‘hotspots’ in p53—mutations that occurred much more frequently in some positions than others—and that the mutations occurred relatively late in tumorigenesis, perhaps driving the transition from benign tumors (adenomas) to malignant ones (carcinomas).

## p53 today

By satisfying the two-hit hypothesis, our initial studies provided definitive genetic evidence that p53 was a tumor suppressor gene. It is interesting that even today, functional studies cannot reliably distinguish between tumor suppressor genes and oncogenes, the only definitive way to classify a cancer driver gene is through genetic approaches (Vogelstein et al., 2013). And now that extensive genome-wide sequencing of human cancers has provided an unbiased view of the mutation landscape, we know that *p53* is the most commonly mutated gene in human cancer. With >28000 mutations reported (p53.IARC.fr), missense mutation remains the most common mechanism for p53 inactivation, accounting for almost 75% of all *p53* mutations. We hope that Arnie, on the occasion of his 80th birthday, can derive considerable satisfaction from the fact that his work inspired a revolution in the understanding of human cancer.

*[We apologize to the many scientists who have made important contributions to p53 research that could not be cited in this brief retrospective.]*
